# The levels of BMI and patterns of obesity and overweight during the COVID-19 pandemic: Experience from the Iran STEPs 2021 survey

**DOI:** 10.3389/fendo.2022.1043894

**Published:** 2022-12-01

**Authors:** Shirin Djalalinia, Moein Yoosefi, Sarvenaz Shahin, Erfan Ghasemi, Nazila Rezaei, Naser Ahmadi, Negar Rezaei, Mehrdad Azmin, Shahabeddin Rezaei, Maryam Nasserinejad, Esmaeil Mohammadi, Rosa Haghshenas, Alireza Namazi Shabestari, Hamidreza Jamshidi, Marziyeh Vahid Dastjerdi, Bagher Larijani, Farshad Farzadfar

**Affiliations:** ^1^ Non-Communicable Diseases Research Center, Endocrinology and Metabolism Population Sciences Institute, Tehran University of Medical Sciences, Tehran, Iran; ^2^ Human Nutrition Program, Department of Human Sciences, The Ohio State University, Columbus, OH, United States; ^3^ National Center for Health Insurance Research, Tehran, Iran; ^4^ Department of Pharmacology, Research Institute for Endocrine Sciences, School of Medicine, Shahid Beheshti University of Medical Sciences, Tehran, Iran; ^5^ Department of Obstetrics and Gynecology, Tehran University of Medical Sciences, Tehran, Iran; ^6^ Endocrinology and Metabolism Research Center, Endocrinology and Metabolism Clinical Sciences Institute, Tehran University of Medical Sciences, Tehran, Iran

**Keywords:** obesity, overweight, BMI, STEPs, Iran

## Abstract

**Background:**

Obesity and its increasing burden have become an urgent health problem all over the world. Benefiting from a national representative sample, the present study aimed to estimate the distribution of body mass index (BMI) levels and its association with metabolic and lifestyle risk factors in an Iranian adult population by sex, age, and geographical distribution.

**Methods:**

This study involves a national survey of noncommunicable disease risk factor surveillance (STEPs) in Iran. Through systematic random sampling, in compliance with safety considerations during the COVID-19 pandemic, of the 28,520 adults who gave voluntary consent and included in the study, 27,874 participants completed the questionnaires (step 1), 27,745 individuals were anthropometrically measured (step 2), and 18,119 individuals completed laboratory evaluation (step 3). Anthropometric measurements followed based on standard protocols and by using calibrated instruments.

**Results:**

In 2021, the national prevalence of normal weight, obesity, and overweight/obesity in ≥18-year-old Iranian adults was estimated at 33.61% (95% CI: 32.99–34.23), 24.96% (24.39–25.53), and 63.02% (62.39–63.65), respectively. Compared to women, Iranian men had a lower mean BMI [25.54 (24.95–26.13 vs. 27.6 (27.07–28.12) kg/m^2^] (*p* < 0.001). There was also a significant difference in the national prevalence rate of overweight/obesity [women: 66% (61–71), men: 53% (46–60) (*p* < 0.001)]. The prevalence of obesity was significantly higher in participants suffering from metabolic and lifestyle risk factors. The study of the geographical extent of obesity and overweight shows that compared to national levels, the highest prevalence of being underweight was seen in the southeastern provinces. On the other hand, the highest prevalence of obesity belonged to the northeastern and central provinces. The highest provincial prevalence of obesity was almost 2.5-fold higher than the lowest provincial prevalence.

**Conclusion:**

The study reveals a significant difference between the prevalence of obesity in male and female participants and between different regions of the country. These findings will help policymakers, clinicians, and researchers to more accurately estimate the obesity/overweight problem and to implement more effective interventional programs to promote strategies of prevention and control of weight gain.

## Introduction

High body mass index (BMI) and its increasing burden represent an urgent problem that needs to be properly addressed (1, 2). Its effect as an important risk factor for cardiovascular disease, diabetes, kidney diseases, some types of cancers, and musculoskeletal disorders is discussed in many studies (3, 4).

Recent analyses reveal that obesity is probably the most important of the four prominent global risk factors that fulfill the criteria required by the government in classifying governmental health priorities (1, 3). Based on standard indicators, over the last two decades, the global prevalence of overweight and obesity has doubled such that nearly a third of the world’s population is now considered obese/overweight (1, 2, 5).

Moreover, the importance and priority of management of obesity/overweight as an important risk factor of attributed mortality and morbidity must be considered due to the global COVID-19 pandemic (6).

In Iran, as in many other countries, obesity and overweight are considered as one of the important health priorities (7). Previous accurate studies confirmed that, following a considerable increase through recent decades, the national age-standardized mean BMI reached 27.9 (27.2–28.7) kg/m² in women and 25.9 (25.2–26.5) kg/m² in men. In addition, the prevalence of overweight/obesity is 71.7% (67.9–75.8) and 36.8% (34.1–39.7) in women and 57.1% (53.7–60.6) and 18.4% (16.9–20) in men (8, 9). The mean number of attributable deaths to excess BMI is estimated as 13,000 in men and 17,000 in women (10). Another study in Iran revealed that, excess BMI could be responsible for 39.5% of total deaths (55.0% men). The highest mortality was attributed to ischemic heart diseases (55.7%), followed by stroke (19.3%) and diabetes (12.0%) (11).

The global agenda of risk reduction was developed on the basis of reliable estimations of the levels, trends, and distribution of NCD risk factors—including obesity and overweight—as a supporting document for designing, implementing, and evaluating the National Action Plans at the country level (3, 12, 13). Given the priority of prevention and management of increasing trends in obesity and overweight, especially during the COVID-19 pandemic, policymakers and health managers need scientific evidence to help them in supporting intervention programs and monitoring health indicators (14, 15).

Benefiting from national and subnational representative samples of STEPs survey 2021 (16), the present investigation included valuable experiences and data during the COVID-19 pandemic, and estimated the prevalence of overweight/obesity and the distribution of BMI levels among the Iranian population by sex and age at national and subnational levels.

## Methods

Following the WHO STEPwise approach to NCD risk factor surveillance, the national STEPs survey 2021 was conducted with representative samples from urban and rural areas of Iran (16). The details of the methods and procedures are discussed elsewhere (16, 17). Here, we only point to some essential requirements.

### Sampling

Aimed at addressing the complexities in regional differences and heterogeneous and incommensurable population distributions and financial considerations, we used the systematic cluster classification method combined with different probability sampling methods. Concurrently with the consideration of the representativeness of the samples at the national and subnational levels, the sample size required for evaluating NCDs’ risk factors in each province was calculated using a proportion-to-size method. Following these approaches and benefiting from the national postal code database, 28,584 participants, from 3,176 clusters, were selected from rural and urban areas of the 31 provinces of Iran.

### Inclusion and exclusion criteria

Based on the study protocol of the 2021 STEPs survey, Iranian adults aged above 18 who resided in urban or rural areas of one of the 31 provinces of Iran were eligible for participation to the study. After collecting information from each household regarding age, sex, and other demographics, three sequential levels of data were gathered (1): demographic, epidemiologic, and metabolic and behavioral risk factors (2); physical measurements; and (3) laboratory measurements (only for participants aged ≥25 years old) (17, 18).

Individuals were excluded if they have a severe physical or mental condition that prevented them from answering the questionnaire or being measured, if they were not able to provide laboratory samples, and if they were pregnant. Data from individuals who were eligible and who agreed to participate and thus completed the inform consent form were collected through interview and measurement.

### Questionnaires (step 1)

Based on the WHO STEPwise approach to risk factor surveillance, sampling and examination processes were completed by trained experts. By using the latest standard version of the WHO questionnaire (version 3.2) (19), validated questionnaires containing demographic and epidemiologic characteristics, lifestyle patterns (e.g., nutritional habits, physical activity, smoking, and alcohol consumption), history of metabolic risk factors and treatment, history of injuries and their risk factors, healthcare utilization, and screening programs were used for recording the data. For more accuracy, the questioning guideline was developed and provided for interviewers.

### Physical measurements (step 2)

Following the WHO criteria and using calibrated instruments, trained healthcare experts were involved in measuring the height, weight, waist circumference, hip circumference, blood pressure, and pulse rate of participants ≥18 years old (16, 17).

Using a standard meter stick, height was measured without shoes, while standing against a wall, with heels, hip, and back of the head in a straight line. Wearing light clothing, participants’ weight was measured to the nearest 100 g with a standard digital scale (Inofit) and calibrated with an index scale of 5 kg before use each time the device was moved. Using standard Beurer sphygmomanometers, systolic and diastolic blood pressures were measured thrice at a time interval of 15 min. The mean values of the second and third instances were reported as the final blood pressure.

The BMI was calculated as weight (kg) by squared height (m^2^). The WHO criteria were used in classifying BMI < 18.5, 18.5 ≤ BMI < 25, 25 ≤ BMI < 30, and 30 ≤ BMI as underweight, normal, overweight, and obese, respectively. Obesity was divided into three categories: class I obese (30 ≤ BMI < 35), class II obese (35 ≤ BMI < 40), and class III obese (40 ≤ BMI) (1, 19).

### Laboratory measurements (step 3)

Aimed at targeting the NCDs’ risk factors through biochemical blood and urine tests, using vaccine transfer boxes, the collected samples were stored and transferred at a temperature of 4°C. Benefiting from a detailed time-binding action plan, sample transfer was set at the shortest possible time (not more than 18 h). All processing and analysis requirements were centralized at the central laboratory of the study using unique brands of devices and kits in the NCDRC laboratory in the Non-Communicable Diseases Research Center (NCDRC) of the Endocrinology and Metabolism Population Sciences Institute of Tehran University of Medical Sciences.

### Definitions of variables

Education was assessed based on the number of successfully completed years of schooling at four subcategories [0 (illiterate), 1–6, 7–12, and >12 years]. Principal component analysis was used to calculate the wealth index from household asset data. According to results, participants’ calculated wealth indices were categorized into five quintiles from the poorest (first quintile) to the richest (fifth quintile). Diabetes based on fasting plasma glucose (FPG) was considered as FPG ≥ 126 mg/dl or self-reporting of consumption of oral hypoglycemic agents (OHA) and/or insulin injection. Diabetes based on HbA1c was evaluated as HbA1c ≥ 6.4% or self-reported (OHA and/or insulin injection).

Among participants who were not recognized as diabetic case, pre-diabetes was defined as 100 ≤ FPG < 126 mg/dl or 5.7 ≤ HbA1c < 6.4%. Hypertriglyceridemia was defined as serum triglycerides ≥ 150 mg/dl. The level of low-density lipoprotein cholesterol ≥ 100 mg/dl considered as criteria for LDL-C detection. Hypercholesterolemia was calculated based on total cholesterol ≥ 200 mg/dl or self-reported related drug consumption. Hypertension criteria were systolic blood pressure ≥ 140 mmHg, a diastolic blood pressure ≥ 90 mmHg, or self-reported related drug consumption. Among participants who were not recognized as hypertension cases, pre-hypertension was detected based on 120 ≤ systolic blood pressure < 140 mmHg or 80 ≤ diastolic blood pressure < 90 mmHg.

### Statistical analyses

To address the problems associated with paper questionnaires, such as missing data and unacceptable data, similar to the previous round in 2016, benefiting from web-based infrastructures, the data were collected through electronic questionnaires and *via* tablets. For higher-quality data cleaning and analysis, all of the processes were conducted by two independent biostatisticians and discrepancies were solved by a third expert.

In the present study, descriptive statistics of variables of interest were presented by sex, age subgroups, and geographical distribution at national and subnational levels. The prevalence rates and means (for all ages) have been presented with 95% confidence interval (95% CI) in the tables. Age standardization of provincial mean BMI and prevalence of each BMI for defined categories was achieved according to the National Population and Housing Census conducted by Iran’s Statistical Center. The results have been presented in maps, with a combination of area of residence and sex. The age-adjusted odds ratio (OR) of BMI categories with respect to smoking status, anthropometry (pre-hypertension and hypertension), and laboratory (pre-diabetes, diabetes, LDL-C, hypertriglyceridemia, and hypercholesterolemia) and self-reported incidence of cardiovascular disease (heart attack and stroke) variables were calculated by logistic regression at three significance levels. These analyses were performed by Stata software (version 11) and R software (version 4.0.2).

### Ethical considerations

All participants were informed about the methods and goals of the study. Participation in survey was voluntary and written informed consent was obtained from all of the participants. The final dataset was de-identified for analysis. Only the primary investigator and the database manager had access to the survey database. The ethical approval for the study was obtained from the ethical committee of the National Institute for Health Research (ID: IR.TUMS.NIHR.REC.1398.006). Moreover, strict COVID-19 preventive guidelines were implemented for all of the participants and those who were involved in the survey/data-gathering step for the duration of the survey during the pandemic (16).

## Results

In the STEPs study 2021, of the 28,520 adults who gave voluntary consent and included in the study, 27,874 participants completed the questionnaires (step 1), 27,745 individuals were anthropometrically measured (step 2), and 18,119 participants completed laboratory evaluation (step 3).

The present study included 73,738 participants (41,025 women and 32,713 men) who had non-missing BMI values. Most of the participants were urban residents (74.97%), aged 25–34 years (23.7%), educated (14.6% were illiterate), married (76.63%), and covered by basic insurance (89.68%). The demographic characteristics of the participants are presented in **Table 1**.

**Table 1:** Demographic characteristics of study participants according to study steps, sex, and age groups.

Age groups	Questionnaires (Step 1)		Physical measurements (Step 2)		Biological assessment (Step 3)	
	Male	Female	Male	Female	Male	Female
**18–24**	1,267	1,405	1,257	1,398	0	0
**25–34**	2,330	2,913	2,314	2,902	1,472	2,077
**35–44**	2,606	3,572	2,590	3,561	1,814	2,649
**45–54**	2,310	3,102	2,294	3,088	1,693	2,403
**55–64**	2,051	2,469	2,042	2,461	1,501	1,811
**65–69**	1,291	1,449	1,287	1,444	922	1,037
**≥70**	624	485	624	483	424	316
**Total**	**12,479**	**15,395**	**12,408**	**15,337**	**7,826**	**10,293**

In 2021, the national prevalence of normal weight, obesity, and overweight/obesity in ≥18-year-old Iranian adults was estimated at 33.61% (95% CI: 32.99–34.23), 24.96% (24.39–25.53), and 63.02% (62.39–63.65), respectively. In the obese group, the distribution of the three obesity categories was estimated at 72.78% (71.68–74.05) in class I obese, 20.83% (19.66–21.82) in class II obese, and 6.39% (5.73–7.05) in class III obese (**Table 2**).

**Table 2:** Distribution of different categories of BMI prevalence (%) according to selected characteristics of Iranian adults.

Variable	Characteristics	Underweight (BMI < 18.5)	Normal (18.5 ≤ BMI < 25)	Overweight (25 ≤ BMI < 30)	Overweight and Obesity (25 ≤ BMI)	Obesity (30 ≤ BMI)	Class I obesity (30 ≤ BMI < 35)	Class II obesity (35 ≤ BMI < 40)	Class III obesity (40 ≤ BMI)
**Overall**		3.37 (3.14,3.6)	33.61 (32.99,34.23)	38.06 (37.42,38.69)	63.02 (62.39,63.65)	24.96 (24.39,25.53)	18.19 (17.68,18.7)	5.18 (4.88,5.47)	1.60 (1.43,1.76)
**Area of residency**	**Rural**	5.62 (5.05,6.19)	37.79 (36.57,39.02)	33.69 (32.49,34.89)	56.58 (55.34,57.83)	22.89 (21.82,23.97)	17.16 (16.19,18.13)	4.34 (3.82,4.86)	1.39 (1.08,1.71)
	**Urban**	2.61 (2.37,2.85)	32.21 (31.49,32.93)	39.52 (38.76,40.27)	65.17 (64.44,65.9)	25.66 (24.99,26.33)	18.54 (17.94,19.13)	5.46 (5.11,5.81)	1.66 (1.46,1.86)
**Sex**	**Female**	3.2 (2.89,3.5)	28.76 (27.96,29.56)	36.82 (35.97,37.68)	68.04 (67.22,68.86)	31.22 (30.4,32.04)	21.63 (20.9,22.36)	7.2 (6.73,7.66)	2.4 (2.12,2.67)
	**Male**	3.58 (3.23,3.94)	39.63 (38.67,40.59)	39.58 (38.62,40.54)	56.79 (55.82,57.76)	17.21 (16.46,17.95)	13.93 (13.25,14.61)	2.68 (2.36,2.99)	0.6 (0.45,0.76)
**Age (years)**	**18 to 24**	12.48 (11.08,13.88)	55.79 (53.67,57.91)	23.39 (21.57,25.21)	31.73 (29.74,33.72)	8.34 (7.17,9.51)	6.76 (5.69,7.84)	1.09 (0.69,1.49)	0.49 (0.19,0.78)
	**25 to 34**	3.94 (3.37,4.51)	44.41 (42.9,45.93)	35.12 (33.66,36.57)	51.65 (50.12,53.17)	16.53 (15.4,17.67)	12.53 (11.52,13.55)	3.01 (2.49,3.53)	0.98 (0.68,1.29)
	**35 to 44**	1.99 (1.6,2.37)	30.95 (29.66,32.23)	41.09 (39.72,42.47)	67.07 (65.76,68.37)	25.97 (24.75,27.2)	19.16 (18.07,20.26)	5.3 (4.67,5.93)	1.51 (1.16,1.87)
	**45 to 54**	1.67 (1.31,2.02)	23.61 (22.34,24.87)	41.49 (40.02,42.97)	74.73 (73.44,76.02)	33.23 (31.82,34.65)	23.6 (22.33,24.86)	7.24 (6.44,8.03)	2.4 (1.94,2.86)
	**55 to 64**	1.73 (1.33,2.13)	25.32 (23.91,26.72)	41.24 (39.64,42.84)	72.95 (71.52,74.39)	31.71 (30.2,33.23)	21.96 (20.61,23.3)	7.39 (6.53,8.25)	2.37 (1.86,2.88)
	**65 to 74**	2.43 (1.79,3.07)	29.79 (27.86,31.72)	39.39 (37.34,41.44)	67.78 (65.81,69.75)	28.39 (26.49,30.29)	21.49 (19.75,23.23)	5.67 (4.7,6.63)	1.23 (0.78,1.68)
	**75 and more**	4.63 (3.25,6.02)	39.87 (36.64,43.11)	35.5 (32.33,38.67)	55.49 (52.2,58.78)	19.99 (17.29,22.69)	15.2 (12.76,17.64)	3.59 (2.36,4.83)	1.2 (0.48,1.92)
**Education**	**Illiterate**	3.97 (3.31,4.63)	31.75 (30.13,33.37)	35.37 (33.71,37.04)	64.28 (62.61,65.94)	28.9 (27.29,30.51)	19.46 (18.05,20.86)	7.11 (6.16,8.05)	2.34 (1.79,2.88)
	**1–6 years**	2.54 (2.13,2.94)	27.41 (26.22,28.6)	36.88 (35.58,38.17)	70.05 (68.83,71.27)	33.17 (31.91,34.44)	23.66 (22.52,24.81)	7.32 (6.61,8.02)	2.19 (1.79,2.59)
	**7–12 years**	3.55 (3,4.09)	32.03 (30.63,33.44)	38.25 (36.77,39.73)	64.42 (62.98,65.86)	26.17 (24.83,27.5)	19.05 (17.85,20.24)	5.54 (4.83,6.25)	1.58 (1.2,1.96)
	**More than 12**	3.52 (3.15,3.88)	38.26 (37.28,39.25)	39.4 (38.41,40.39)	58.22 (57.22,59.21)	18.82 (18.02,19.61)	14.45 (13.73,15.16)	3.32 (2.95,3.68)	1.05 (0.84,1.27)
**Marital status**	**Never married**	9.14 (8.18,10.1)	52.25 (50.56,53.93)	27.44 (25.93,28.96)	38.62 (36.97,40.26)	11.17 (10.11,12.24)	8.8 (7.84,9.76)	1.57 (1.17,1.97)	0.8 (0.5,1.11)
	**Married**	2.24 (2.03,2.46)	30.44 (29.75,31.13)	40.32 (39.58,41.06)	67.32 (66.62,68.02)	26.99 (26.32,27.66)	19.67 (19.08,20.27)	5.66 (5.3,6.01)	1.66 (1.47,1.86)
	**Divorced/separated**	4.71 (2.82,6.59)	34.89 (30.63,39.15)	39.2 (34.79,43.62)	60.41 (56.02,64.79)	21.2 (17.47,24.94)	15.66 (12.33,19)	3.76 (2.04,5.48)	1.78 (0.56,3)
	**Widowed**	2.83 (1.98,3.68)	27.14 (24.8,29.48)	35.35 (32.83,37.86)	70.03 (67.62,72.43)	34.68 (32.16,37.21)	23.52 (21.26,25.77)	8.54 (7.05,10.04)	2.62 (1.76,3.48)
**Basic insurance**	**No**	3.66 (2.89,4.43)	36.69 (34.68,38.7)	37.18 (35.17,39.19)	59.65 (57.61,61.69)	22.48 (20.73,24.22)	16.33 (14.79,17.88)	4.77 (3.88,5.66)	1.37 (0.87,1.88)
	**Yes**	3.32 (3.08,3.56)	33.26 (32.61,33.91)	38.14 (37.46,38.82)	63.42 (62.76,64.09)	25.29 (24.68,25.89)	18.41 (17.87,18.95)	5.25 (4.94,5.57)	1.62 (1.44,1.8)
**Complementary insurance**	**No**	4.04 (3.75,4.34)	35.62 (34.87,36.36)	37.02 (36.27,37.77)	60.34 (59.59,61.1)	23.32 (22.66,23.98)	17.09 (16.5,17.68)	4.78 (4.44,5.11)	1.46 (1.27,1.65)
	**Yes**	1.62 (1.31,1.94)	28.48 (27.34,29.61)	40.7 (39.47,41.94)	69.9 (68.75,71.06)	29.2 (28.05,30.34)	20.96 (19.94,21.98)	6.31 (5.68,6.93)	1.93 (1.58,2.28)
**Wealth index quintile**	**Poorest**	6.1 (5.39,6.81)	38.81 (37.34,40.29)	33.39 (31.96,34.82)	55.09 (53.59,56.59)	21.7 (20.44,22.96)	15.33 (14.23,16.42)	4.82 (4.16,5.49)	1.55 (1.16,1.94)
	**2**	3.62 (3.09,4.15)	34.5 (33.08,35.92)	36.71 (35.26,38.16)	61.88 (60.43,63.32)	25.17 (23.85,26.48)	17.94 (16.78,19.1)	5.29 (4.6,5.99)	1.93 (1.5,2.36)
	**3**	2.9 (2.39,3.42)	32.99 (31.56,34.43)	37.85 (36.36,39.34)	64.11 (62.64,65.57)	26.26 (24.91,27.61)	19.59 (18.37,20.81)	5.23 (4.54,5.91)	1.45 (1.08,1.81)
	**4**	2.38 (1.92,2.84)	31.17 (29.76,32.58)	39.61 (38.13,41.1)	66.45 (65.01,67.89)	26.83 (25.49,28.18)	20.15 (18.93,21.36)	5.25 (4.57,5.92)	1.44 (1.08,1.8)
	**Richest**	1.69 (1.31,2.06)	31.93 (30.54,33.32)	42.62 (41.14,44.1)	66.38 (64.97,67.79)	23.76 (22.49,25.03)	17.18 (16.06,18.31)	5.15 (4.48,5.82)	1.42 (1.07,1.78)

Data in parentheses are 95% confidence intervals (CI).

Compared to women, Iranian men had a lower mean BMI [25.66 (25.16–26.15) vs. 27.15 (26.53–27.76) kg/m^2^] (*p* < 0.001). There was also a significant difference in the national prevalence rate of overweight/obesity [women: 61.51% (55.92–67.1), men: 54.5% (48.38–60.62) (*p* < 0.001)] (**Table 3**). Another noticeable point is that 5.23% (2.23–8.24) of female participants and 4.46% (1.93–6.99) of male participants were underweight (**Table 3**).

**Table 3:** Distribution of different categories of BMI prevalence (%), by sex and province, 2021.

BMI categories	Age standardized mean BMI (kg/m^2^) (UI)		Underweight (BMI < 18.5)		Normal (18.5 ≤ BMI <25)		Overweight (25 ≤ BMI <30)		Obesity (30 ≤ BMI)		Class I obesity (30 ≤ BMI < 35)		Class II obesity (35 ≤ BMI < 40)		Class III obesity (40 ≤ BMI)	
Province	Female	Male	Female	Male	Female	Male	Female	Male	Female	Male	Female	Male	Female	Male	Female	Male
**National**	27.15 (26.53,27.76)	25.66 (25.16,26.15)	5.23 (2.23,8.24)	4.46 (1.93,6.99)	33.25 (27.56,38.95)	41.04 (34.96,47.11)	32.64 (27.08,38.2)	40.18 (34.16,46.21)	28.87 (23.76,33.98)	14.32 (10.31,18.33)	21.44 (16.69,26.19)	11.74 (8.06,15.42)	5.61 (3.34,7.89)	2.58 (0.75,4.41)	1.81 (0.24,3.38)	0 (0,0)
**Markazi**	28.08 (27.47,28.7)	26.66 (25.75,27.57)	3.26 (1.12,5.4)	6.14 (1.71,10.57)	28.96 (23.57,34.35)	33.56 (25.88,41.24)	30.48 (25.11,35.85)	39.3 (31.47,47.12)	37.3 (31.86,42.74)	21.01 (14.4,27.61)	25.88 (20.82,30.95)	14.5 (9.17,19.84)	7.84 (4.99,10.68)	4.75 (0.98,8.52)	3.58 (1.7,5.47)	1.75 (−0.62,4.13)
**Gilan**	27.85 (27.33,28.36)	26.98 (26.33,27.62)	2.59 (0.58,4.59)	0.96 (−0.26,2.18)	30.81 (25.92,35.7)	38.96 (33.2,44.72)	35.95 (30.97,40.93)	35.85 (30.34,41.37)	30.66 (26.24,35.07)	24.23 (19.2,29.25)	20.3 (16.26,24.34)	18.58 (14.13,23.04)	7.97 (5.53,10.4)	3.05 (1.05,5.04)	2.39 (1.25,3.53)	2.6 (0.45,4.75)
**Mazandaran**	27.65 (27.18,28.12)	25.94 (25.48,26.4)	2.86 (0.98,4.74)	2.04 (0.44,3.65)	30.1 (25.63,34.57)	44.92 (39.74,50.11)	35.26 (30.66,39.87)	34.92 (29.95,39.89)	31.78 (27.8,35.77)	18.11 (14.31,21.91)	22.44 (18.74,26.15)	13.82 (10.38,17.27)	8.18 (5.94,10.41)	3.52 (1.73,5.3)	1.16 (0.35,1.98)	0.77 (−0.02,1.56)
**Azerbaijan, East**	27.85 (27.38,28.31)	26.2 (25.71,26.68)	2.73 (1.03,4.44)	2.06 (0.39,3.73)	30.04 (25.7,34.38)	37.85 (32.53,43.17)	32.37 (27.81,36.94)	42.55 (37.04,48.06)	34.85 (30.54,39.16)	17.54 (13.35,21.72)	27 (22.81,31.2)	13.78 (10.02,17.54)	6.31 (4.21,8.4)	3.37 (1.4,5.34)	1.54 (0.53,2.55)	0.38 (−0.36,1.13)
**Azerbaijan, West**	26.56 (26.12,27)	25.88 (25.43,26.33)	3.81 (1.8,5.82)	3.64 (1.74,5.55)	34.71 (30.26,39.17)	39.27 (33.99,44.54)	37.8 (33.21,42.38)	43.99 (38.57,49.42)	23.68 (19.96,27.4)	13.1 (9.41,16.78)	18.42 (15.06,21.78)	9.89 (6.63,13.14)	4.06 (2.34,5.78)	1.95 (0.66,3.25)	1.2 (0.31,2.1)	1.26 (−0.23,2.74)
**Kermanshah**	27.95 (27.41,28.5)	26.05 (25.63,26.47)	4.49 (2.66,6.32)	3.04 (1.3,4.78)	30.73 (26.51,34.96)	39.82 (35.02,44.61)	33.14 (28.94,37.34)	39.97 (35.22,44.73)	31.64 (27.68,35.59)	17.17 (13.73,20.6)	19.02 (15.55,22.48)	13.77 (10.61,16.93)	7.69 (5.48,9.9)	2.8 (1.29,4.3)	4.93 (3.11,6.75)	0.6 (−0.07,1.27)
**Khuzestan**	26.65 (26.22,27.08)	25.1 (24.67,25.53)	5.07 (3.02,7.12)	6.99 (4.51,9.48)	33.96 (29.93,38)	44.48 (39.79,49.16)	36.62 (32.56,40.69)	35.2 (30.77,39.63)	24.34 (21.12,27.57)	13.33 (10.26,16.4)	17.32 (14.47,20.18)	11.41 (8.58,14.25)	5.61 (3.85,7.37)	0.99 (−0.03,2.01)	1.41 (0.45,2.37)	0.93 (0.07,1.79)
**Fars**	26.32 (25.72,26.91)	23.93 (23.44,24.41)	7.12 (4.41,9.82)	10.72 (7.3,14.13)	37.17 (32.22,42.12)	51.75 (46.31,57.2)	31.66 (26.88,36.44)	28.69 (23.89,33.49)	24.05 (19.7,28.41)	8.84 (5.65,12.04)	17.42 (13.53,21.32)	6.86 (3.98,9.75)	4.06 (2.04,6.08)	1.98 (0.49,3.47)	2.57 (0.86,4.28)	0 (0,0)
**Kerman**	27.01 (26.64,27.39)	25.23 (24.88,25.58)	5.03 (3.24,6.82)	5.15 (3.29,7)	30.35 (26.83,33.87)	44.21 (40.18,48.24)	38.24 (34.57,41.92)	36.09 (32.29,39.89)	26.37 (23.32,29.42)	14.55 (11.76,17.34)	19.91 (17.1,22.71)	12.8 (10.14,15.47)	4.84 (3.41,6.27)	1.63 (0.66,2.61)	1.62 (0.78,2.45)	0.11 (−0.11,0.33)
**Khorasan, Razavi**	26.78 (26.32,27.24)	25.66 (25.25,26.06)	5.72 (3.52,7.92)	4.12 (2.23,6)	36.01 (32.03,39.99)	41.11 (36.45,45.77)	33.24 (29.48,37.01)	39.05 (34.47,43.63)	25.03 (21.81,28.25)	15.72 (12.59,18.86)	17.37 (14.6,20.13)	12.72 (9.81,15.63)	4.97 (3.49,6.45)	3 (1.6,4.4)	2.69 (1.27,4.12)	0 (0,0)
**Isfahan**	24.43 (24.01,24.85)	23.35 (22.91,23.8)	13.21 (10.36,16.05)	14.3 (10.92,17.69)	45.18 (40.96,49.39)	50.8 (45.91,55.7)	28.24 (24.5,31.99)	24.19 (20.01,28.37)	13.38 (10.61,16.14)	10.7 (7.59,13.82)	9.93 (7.47,12.38)	10.44 (7.36,13.52)	2.22 (1.02,3.42)	0.27 (−0.25,0.79)	1.23 (0.32,2.15)	0 (0,0)
**Sistan and Baluchistan**	28.25 (27.75,28.75)	26.31 (25.78,26.83)	3.87 (1.76,5.98)	2.66 (0.84,4.49)	23.36 (19.09,27.62)	37 (31.45,42.55)	37.03 (32.28,41.78)	40.36 (34.83,45.89)	35.74 (31.15,40.33)	19.99 (15.49,24.48)	25.16 (20.96,29.37)	17.29 (13.03,21.54)	8.29 (5.6,10.98)	2.27 (0.75,3.78)	2.29 (0.92,3.65)	0.43 (−0.41,1.27)
**Kordestan**	26.98 (26.5,27.46)	25.1 (24.55,25.65)	5.23 (2.78,7.68)	4.91 (2.3,7.52)	35.04 (30.33,39.75)	50.52 (44.44,56.61)	32.03 (27.46,36.6)	32.1 (26.74,37.46)	27.71 (23.74,31.68)	12.47 (8.32,16.61)	19.63 (16.06,23.2)	10.05 (6.34,13.77)	6.41 (4.24,8.58)	1.76 (−0.18,3.7)	1.66 (0.47,2.86)	0.66 (−0.18,1.49)
**Hamadan**	26.56 (26.14,26.99)	25.63 (25.11,26.15)	3.29 (1.41,5.18)	5.61 (2.73,8.49)	38.58 (34.1,43.07)	41.62 (35.99,47.25)	36.03 (31.62,40.45)	37.9 (32.47,43.34)	22.09 (18.59,25.59)	14.87 (10.95,18.8)	15.94 (12.8,19.07)	11.38 (7.93,14.84)	4.6 (2.86,6.34)	3.2 (1.15,5.24)	1.55 (0.43,2.68)	0.29 (−0.11,0.69)
**Chahar Mahaal and Bakhtiari**	26.7 (26.25,27.14)	25.93 (25.4,26.47)	2.85 (1.2,4.49)	2.54 (0.81,4.28)	35.28 (30.59,39.97)	43.59 (37.81,49.37)	38.75 (34.03,43.48)	38.23 (32.59,43.88)	23.11 (19.21,27.01)	15.63 (11.46,19.81)	18.99 (15.24,22.74)	11.21 (7.62,14.8)	3.13 (1.77,4.49)	3.42 (1.36,5.48)	0.99 (0.21,1.78)	1.01 (−0.24,2.25)
**Lorestan**	26.93 (26.32,27.55)	25.88 (25.45,26.32)	3.05 (0.53,5.58)	0.26 (−0.23,0.74)	35.16 (29.21,41.1)	42.47 (36.38,48.55)	34.81 (28.97,40.65)	44.54 (38.43,50.66)	26.98 (22.09,31.87)	12.73 (8.76,16.71)	21.15 (16.63,25.67)	11.1 (7.32,14.87)	4.27 (2.05,6.49)	1.64 (0.16,3.11)	1.56 (0.08,3.05)	0 (0,0)
**Ilam**	28.12 (27.56,28.69)	25.42 (24.97,25.88)	1.63 (0.22,3.03)	2.47 (0.66,4.28)	28.37 (23.5,33.23)	46.74 (41.07,52.4)	35.25 (30.01,40.49)	38 (32.52,43.48)	34.75 (29.88,39.62)	12.79 (8.95,16.63)	21.44 (17.14,25.75)	11.6 (7.96,15.23)	11.03 (7.81,14.25)	0.89 (−0.33,2.11)	2.28 (0.89,3.68)	0.3 (−0.29,0.89)
**Kohgiluyeh and Boyer-Ahmad**	27.52 (27.05,27.98)	25.81 (25.36,26.26)	2.42 (0.94,3.91)	4.58 (2.54,6.62)	32.94 (28.99,36.88)	43.98 (39.29,48.68)	35.69 (31.69,39.69)	34.58 (30.17,39)	28.94 (25.18,32.71)	16.86 (13.32,20.39)	20.83 (17.43,24.22)	12.33 (9.25,15.42)	4.79 (3.04,6.54)	3.54 (1.84,5.23)	3.33 (1.93,4.73)	0.98 (−0.11,2.08)
**Bushehr**	27.15 (26.75,27.56)	25.35 (24.91,25.78)	3.82 (1.63,6)	4.87 (2.24,7.5)	29.88 (25.62,34.14)	42.48 (37.27,47.69)	40.32 (36.01,44.63)	39.48 (34.47,44.49)	25.98 (22.43,29.53)	13.17 (10.02,16.32)	18.32 (15.16,21.47)	11.18 (8.22,14.15)	6.67 (4.61,8.73)	1.75 (0.59,2.9)	1 (0.31,1.69)	0.24 (−0.23,0.71)
**Zanjan**	27.23 (26.67,27.78)	26.01 (25.47,26.55)	2.73 (0.75,4.72)	3.87 (1.18,6.55)	30.58 (24.98,36.17)	36.87 (30.82,42.93)	39.2 (33.35,45.06)	41.61 (35.69,47.53)	27.49 (22.81,32.17)	17.65 (13,22.3)	20.89 (16.43,25.35)	13.9 (9.68,18.12)	4.84 (2.94,6.75)	3.74 (1.42,6.07)	1.75 (0.59,2.92)	0 (0,0)
**Semnan**	27.44 (26.89,27.99)	26 (25.49,26.51)	5.81 (3.17,8.45)	4.46 (2.39,6.53)	29.53 (24.93,34.13)	40.11 (35.28,44.95)	34.07 (29.43,38.7)	38.24 (33.5,42.99)	30.6 (26.22,34.98)	17.18 (13.5,20.86)	20.16 (16.26,24.06)	12.44 (9.27,15.62)	8.4 (5.85,10.94)	3.86 (1.95,5.78)	2.04 (0.88,3.2)	0.87 (−0.12,1.87)
**Yazd**	25.21 (24.65,25.78)	24.97 (24.11,25.83)	11.94 (8.2,15.68)	6.19 (3.1,9.28)	34.96 (29.28,40.64)	51.68 (44.73,58.62)	35.01 (28.93,41.09)	29.95 (23.47,36.43)	18.09 (14.15,22.02)	12.18 (7.3,17.06)	13.69 (10.14,17.23)	9.43 (5,13.87)	3.52 (1.55,5.49)	1.58 (−0.25,3.4)	0.88 (−0.12,1.88)	1.17 (−0.82,3.15)
**Hormozgān**	26.95 (26.71,27.19)	26.31 (26.07,26.55)	1.97 (1.01,2.94)	1.52 (0.71,2.32)	37.47 (34.98,39.97)	37.48 (34.63,40.33)	36.88 (34.41,39.35)	44.13 (41.24,47.01)	23.67 (21.73,25.61)	16.87 (14.7,19.05)	16.3 (14.62,17.99)	13.48 (11.49,15.47)	5.72 (4.62,6.82)	2.81 (1.87,3.75)	1.65 (1.06,2.24)	0.58 (0.15,1.01)
**Tehran**	27.7 (27.15,28.24)	26.36 (25.73,27)	1.63 (−0.12,3.38)	3.42 (0.34,6.5)	29.02 (23.72,34.32)	34.26 (27.55,40.97)	39.27 (33.54,45.01)	45.5 (38.72,52.27)	30.08 (25.22,34.93)	16.83 (12.13,21.52)	21.22 (16.91,25.53)	12.84 (8.68,17.01)	7.53 (4.66,10.4)	3.45 (1.08,5.81)	1.33 (0.05,2.61)	0.54 (−0.23,1.3)
**Ardabil**	27.33 (26.76,27.91)	27.08 (26.45,27.72)	3.19 (0.91,5.47)	2.73 (0.54,4.91)	33.99 (28.18,39.8)	35.59 (29.56,41.62)	33.04 (27.38,38.7)	38.56 (32.46,44.66)	29.78 (24.71,34.86)	23.12 (18.12,28.13)	20.76 (16.14,25.38)	15.81 (11.42,20.19)	6.89 (4.46,9.31)	5.52 (2.78,8.25)	2.14 (0.44,3.84)	1.8 (0.15,3.45)
**Qom**	27.91 (27.34,28.49)	25.52 (24.96,26.07)	1.7 (0.16,3.25)	2.44 (0.21,4.67)	28.32 (23.1,33.54)	45.86 (39.48,52.24)	40.26 (34.63,45.89)	35.79 (29.84,41.74)	29.72 (24.96,34.47)	15.91 (11.26,20.55)	18.33 (14.35,22.31)	12.88 (8.75,17.01)	8.02 (5.02,11.01)	3.03 (0.69,5.37)	3.37 (1.58,5.16)	0 (0,0)
**Qazvin**	27.06 (26.57,27.54)	26.13 (25.61,26.65)	3.98 (2.13,5.84)	4.46 (2.13,6.78)	33.65 (29.24,38.06)	40.94 (35.88,46.01)	34.83 (30.36,39.29)	33.44 (28.6,38.29)	27.54 (23.67,31.41)	21.16 (17.12,25.19)	19.79 (16.33,23.25)	16.99 (13.31,20.68)	6.03 (3.92,8.14)	3.18 (1.36,5)	1.72 (0.58,2.86)	0.98 (−0.13,2.1)
**Golestan**	26.97 (26.45,27.49)	25.75 (25.25,26.24)	3.21 (1.45,4.96)	4.42 (2.07,6.77)	34.08 (29.54,38.62)	42.89 (37.62,48.17)	35.85 (31.17,40.52)	33.52 (28.43,38.61)	26.86 (22.78,30.95)	19.16 (14.98,23.34)	16.94 (13.44,20.43)	15.64 (11.76,19.52)	7.87 (5.33,10.42)	2.95 (1.16,4.75)	2.05 (0.69,3.42)	0.57 (−0.23,1.36)
**Khorasan, North**	25.5 (24.99,26)	24.71 (23.98,25.44)	6.33 (3.7,8.95)	7.63 (4.15,11.11)	43.11 (37.84,48.37)	51.07 (44.55,57.6)	31.57 (26.61,36.53)	26.13 (20.49,31.77)	19 (15.18,22.82)	15.16 (10.38,19.95)	13.58 (10.22,16.95)	11.04 (6.82,15.26)	4.39 (2.34,6.45)	2.18 (0.43,3.94)	1.02 (0.15,1.9)	1.94 (−0.02,3.9)
**Khorasan, South**	26.89 (26.16,27.62)	25.83 (25.31,26.35)	4.78 (1.51,8.05)	0.9 (−0.47,2.27)	37.84 (32.07,43.61)	49.08 (42.82,55.35)	32.54 (27.7,37.37)	35.73 (29.72,41.74)	24.84 (20.1,29.58)	14.29 (10.05,18.54)	15.82 (11.9,19.74)	11.91 (7.96,15.86)	5.65 (3.48,7.83)	1.78 (0.17,3.39)	3.37 (0.96,5.78)	0.6 (−0.23,1.44)
**Alborz**	27.78 (27.68,27.88)	26 (25.91,26.09)	3.2 (2.89,3.5)	3.58 (3.23,3.93)	28.76 (27.96,29.55)	39.62 (38.66,40.58)	36.83 (35.97,37.68)	39.59 (38.62,40.55)	31.22 (30.4,32.04)	17.21 (16.47,17.95)	21.63 (20.9,22.36)	13.93 (13.25,14.62)	7.2 (6.73,7.66)	2.68 (2.36,2.99)	2.4 (2.12,2.67)	0.6 (0.45,0.76)

**Figure 1:**
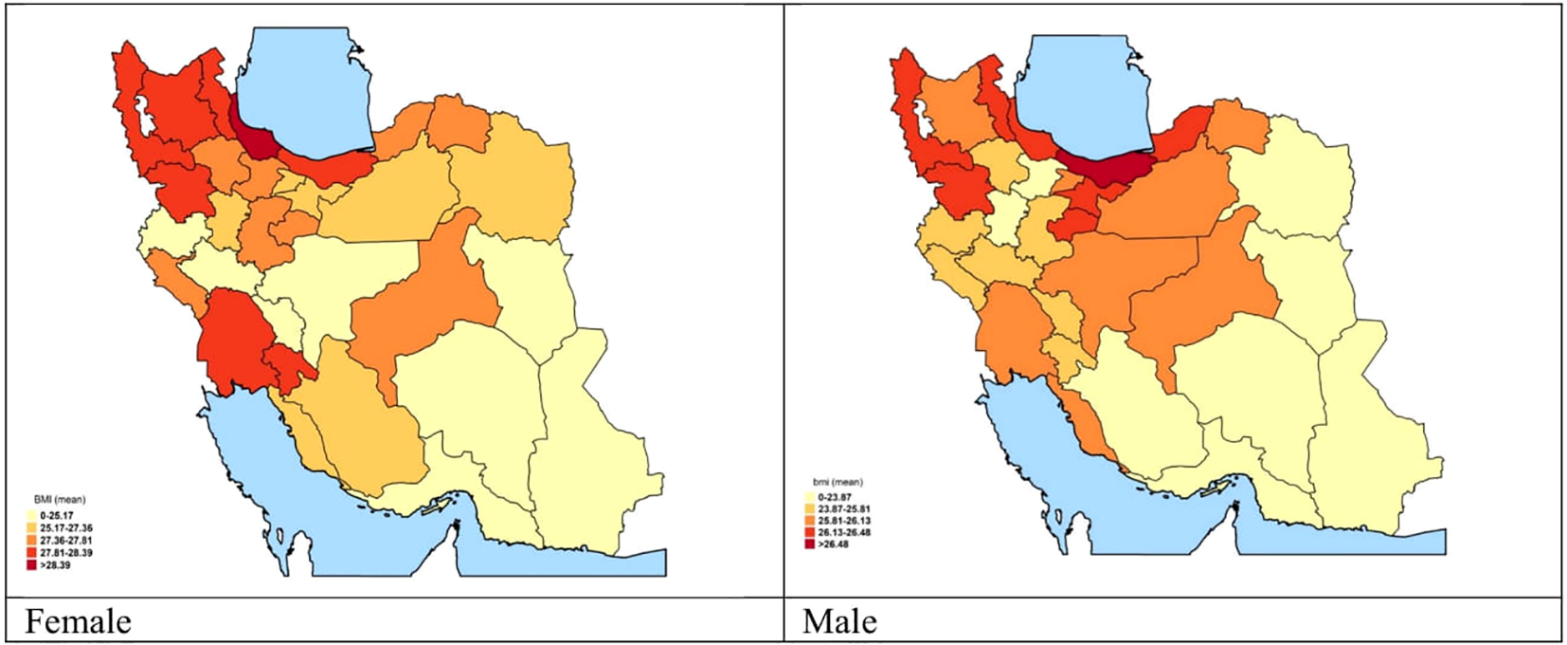
The provincial distribution of Mean BMI by sex.

**Figure 2:**
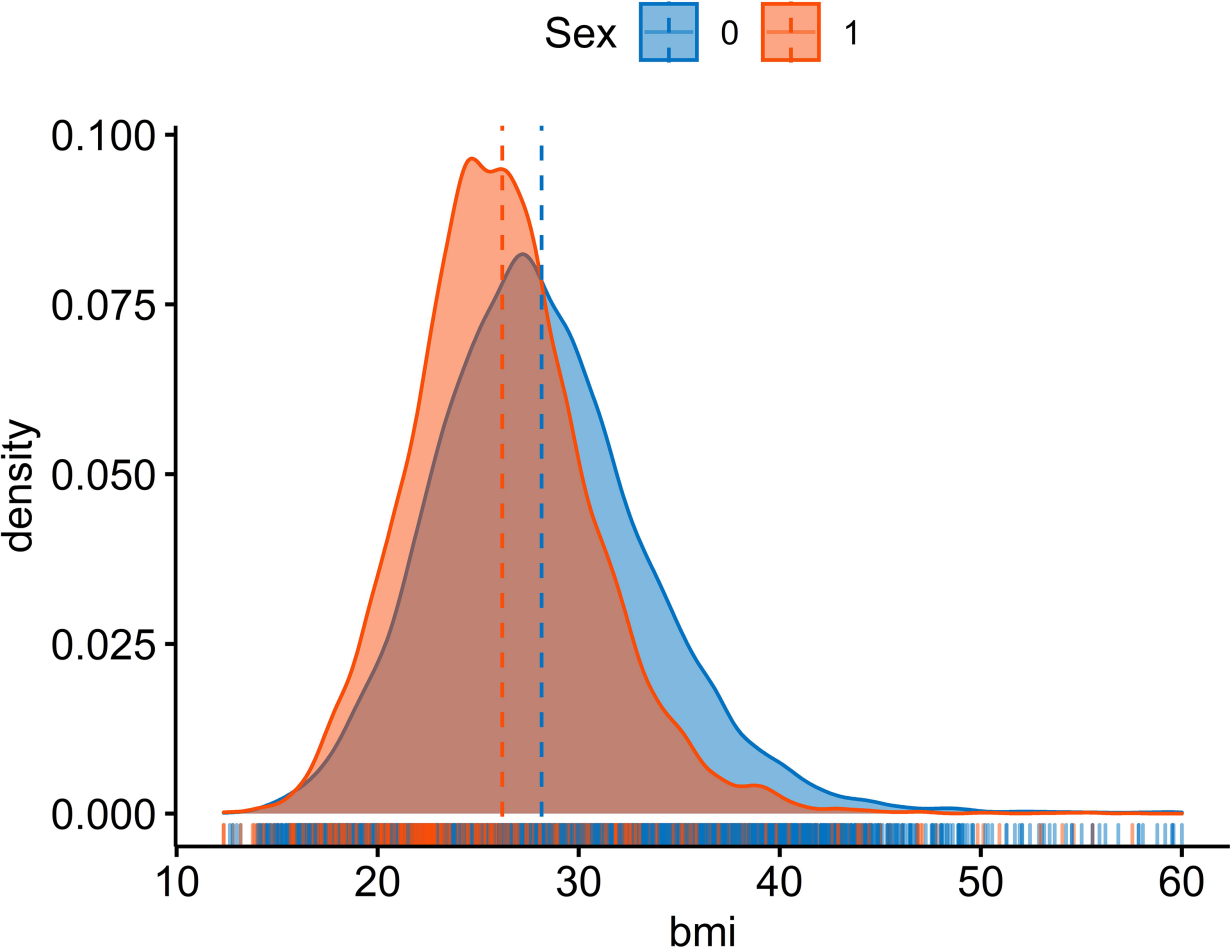
Distribution of Mean BMI by Sex in People Aged 18 Years and Older in 2021..

Comparing BMI distribution in female and male populations, more than the left shift (increase) in the average BMI of both male and female populations, the detected peak in the distribution of BMI especially in female participants is considerable (**Figure 1**). The relation between age-standardized BMI, obesity prevalence, and overweight prevalence in men and women is presented in **Figure 2**.

Given the comparative results of the age groups, the highest and lowest prevalence of obesity belonged to the 45–54 [33.23 (31.82,34.65)] and 18–24 [8.34 (7.17,9.51)] age groups, respectively. In the overweight/obese group, the highest and the lowest estimates belonged to the age groups of 45–54 [74.73 (73.44,76.02)] and 18–24 [31.73 (29.74,33.72)], respectively (**Table 2**).

The analysis of results showed that participants with >12 years of schooling had a significantly lower prevalence of obesity 18.82 (18.02–19.61) (*p* < 0.001). With regard to marriage status, the never married population had the lowest prevalence of both obesity [11.17 (10.11–12.24)] and overweight [27.44 (25.93–28.96)] among all the age groups (**Table 2**).

**Figure 3:**
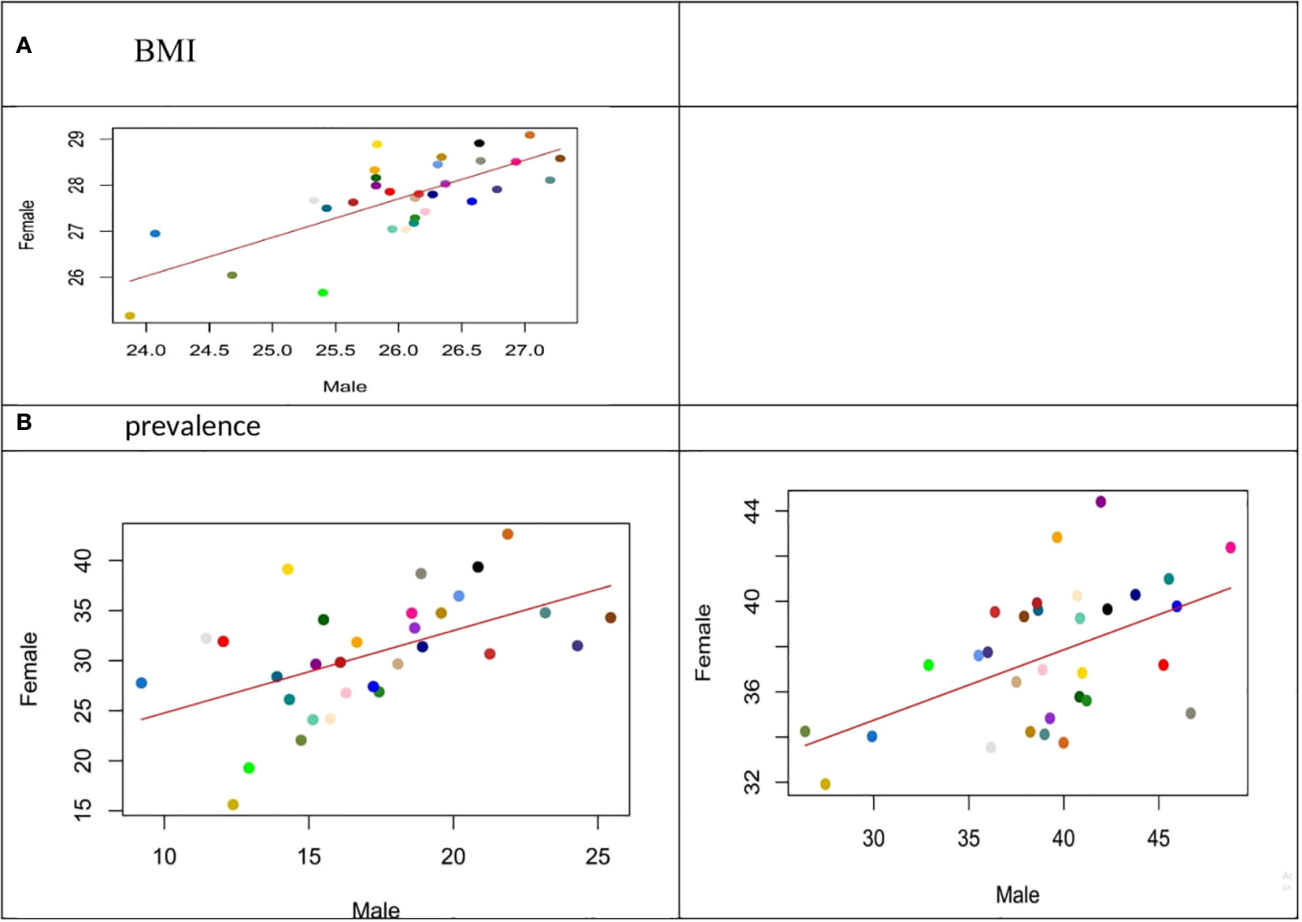
(Scatter Plot): Relation between age-standardized BMI, obesity prevalence and overweight prevalence in men and women aged 18 years and older in 2021.

Based on provincial patterns, the highest prevalence of being underweight was seen in the southeastern provinces. On the other hand, the highest prevalence of obesity belonged to the northeastern and central provinces (**Table 1**, **Figure 3**).

**Figure 4:**
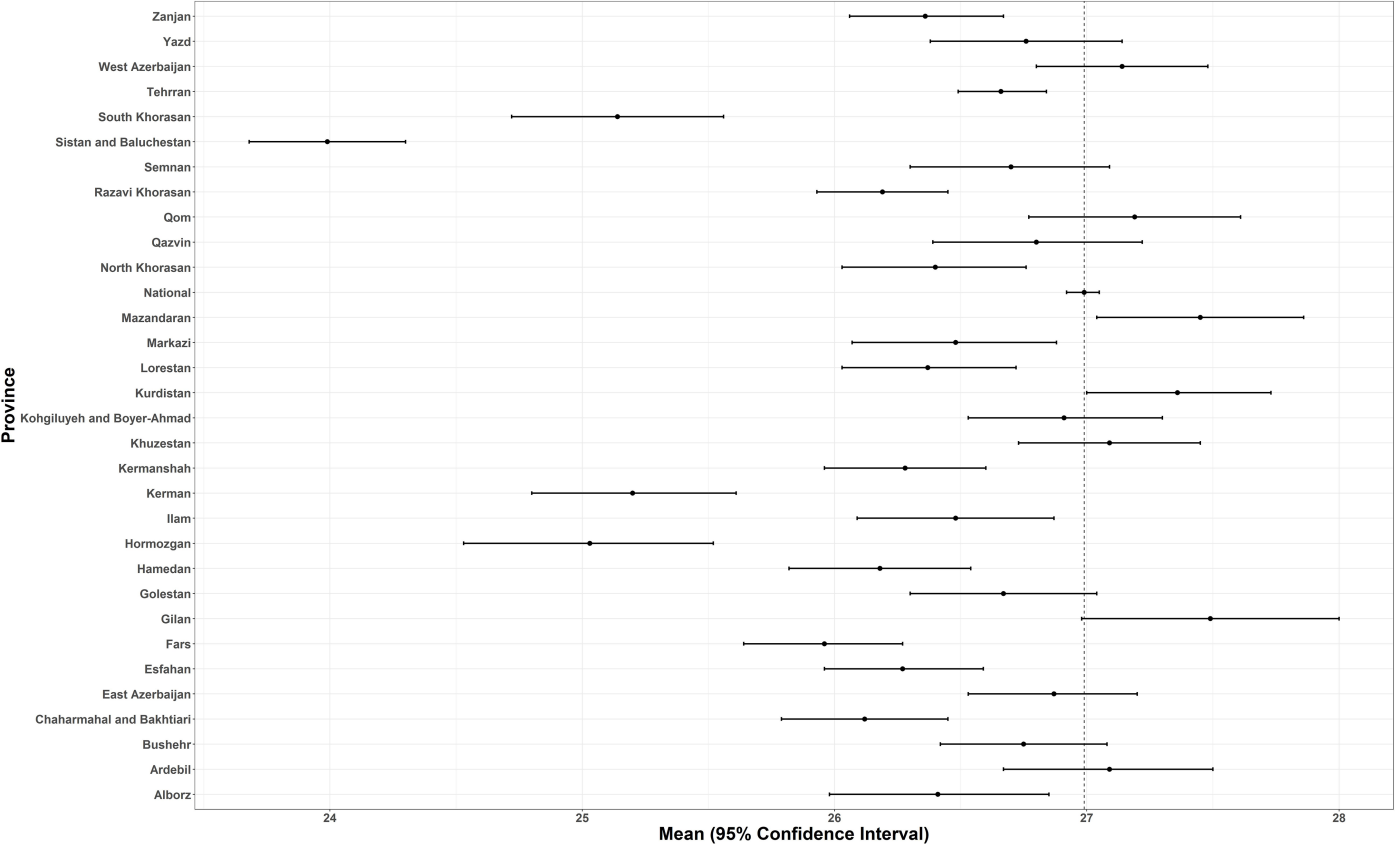
The national and provincial age-standardized mean BMI.

Considering the distribution of different categories of BMI across the provinces, the highest prevalence of obesity belonged to West Azerbaijan’s women and Ardebil’s men [42% (37–47) and 24% (19–30), respectively]. Based on these estimations, Sistan and Baluchistan have the highest prevalence of underweight for both female and male participants [13.24 (9.36–17.11) and 16.22 (12.2–20.25), respectively] (**Table 1**, **Figure 4**).

It is noteworthy that in many provinces such as Kerman, a double burden of obesity was detected, especially among female participants (underweight prevalence is 9% and obesity prevalence is 23% for female participants).

Compared to rural areas, the prevalence of obesity and overweight/obesity significantly was higher among individuals from urban areas for all age groups, in both sex [25.66 (24.99–26.33)] for men and [65.17 (64.44–65.9)] (for women *p* < 0.001) (**Table 3**).

The prevalence of obesity was significantly higher in participants suffering from metabolic and lifestyle risk factors. Among female participants, pre-diabetes based on FPG [OR: 1.68 (1.46–1.93)] or diabetes based on FPG [OR: 2.13 (1.83–2.48)] or HbA1c [OR: 2.49 (2.14–2.90)], hypercholesterolemia [OR: 1.54 (1.36–1.74)], and hypertension [OR: 2.697 (2.49–2.922)] increased the age-adjusted OR of obesity. In male participants, diabetes based on FPG [OR: 2.302 (1.85–2.87)] or HbA1c [(OR: 2.46 (2.01–3.01)], hypercholesterolemia [OR: 1.591 (1.34–1.90)], and hypertension [OR: 2.31 (2.07–2.57)] increased the age-adjusted OR of obesity.

In female participants, the increased risk of age-adjusted overweight/obesity was significantly related to diabetes based on FPG [OR: 2.14 (1.72–2.66)] or HbA1c [OR: 2.63 (2.13–3.24), LDL-C [OR: 1.41 (1.25–1.60)], hypercholesterolemia [OR: 1.81 (1.55–2.11)], hypertension [OR: 2.94 (2.68–3.21)], and prehypertension [OR: 1.45 (1.286–1.635)]. Among male participants, significant associations were confirmed in diabetes based on FPG [OR: 2.081 (1.66–2.61)] or HbA1c [OR: 2.187 (1.79–2.68)], hypercholesterolemia [OR: 1.81 (1.55–2.11)], hypertension [OR: 2.43 (2.21–2.67)], and prehypertension [OR: 1.45 (1.29–1.64)]. Ever tobacco smoking, ever daily cigarette smoking, and current daily cigarette smoking increased the age-adjusted OR of overweight/obesity in male participants (**Table 4**).

**Table 4:** The odds ratio of BMI categories according to metabolic and lifestyle risk factors of Iranian adults by sex.

Variable	Sex	Underweight (BMI < 18.5)	*p*-value	Normal (18.5 ≤ BMI < 25)	*p*-value	Overweight (25 ≤ BMI < 30)	*p*-value	Obesity (30 ≤ BMI)	*p*-value
**Diabetes (FPG ≥ 126 or self-report)**	Female	0.118 (0.052,0.267)	<0.001	0.509 (0.409,0.634)	<0.001	0.81 (0.692,0.948)	0.009	2.128 (1.826,2.481)	<0.001
	Male	0.199 (0.094,0.422)	<0.001	0.527 (0.419,0.662)	<0.001	1.061 (0.875,1.287)	0.548	2.304 (1.85,2.868)	<0.001
**Diabetes (HbA1c ≥ 6.4% or self-report)**	Female	0.072 (0.029,0.177)	<0.001	0.417 (0.338,0.516)	<0.001	0.788 (0.676,0.918)	0.002	2.487 (2.137,2.895)	<0.001
	Male	0.275 (0.153,0.496)	<0.001	0.496 (0.403,0.61)	<0.001	1.067 (0.895,1.272)	0.472	2.463 (2.015,3.01)	<0.001
**Pre-diabetes (100 ≤ FPG < 126)**	Female	0.506 (0.285,0.9)	0.02	0.63 (0.532,0.746)	<0.001	0.886 (0.771,1.018)	0.088	1.681 (1.461,1.933)	<0.001
	Male	0.941 (0.619,1.433)	0.778	0.77 (0.658,0.901)	0.001	1.162 (1,1.35)	0.05	1.178 (0.983,1.41)	0.077
**Pre-diabetes (5.7% ≤ HbA1c < 6.4%)**	Female	0.86 (0.616,1.2)	0.375	0.716 (0.627,0.819)	<0.001	1.13 (1.006,1.269)	0.039	1.167 (1.039,1.312)	0.009
	Male	1.38 (0.994,1.916)	0.054	0.932 (0.818,1.062)	0.289	1.043 (0.916,1.188)	0.528	0.985 (0.835,1.163)	0.862
**LDL-C** **(LDL ≥ 100 mg/dl)**	Female	0.601 (0.431,0.838)	0.003	0.73 (0.643,0.829)	<0.001	1.161 (1.036,1.302)	0.01	1.155 (1.028,1.297)	0.015
	Male	0.463 (0.319,0.671)	<0.001	0.986 (0.865,1.123)	0.829	1.135 (0.997,1.293)	0.056	0.945 (0.799,1.116)	0.502
**Hypercholesterolemia (total cholesterol ≥ 200 mg/dl or self-report of drug taking)**	Female	0.381 (0.254,0.572)	<0.001	0.577 (0.497,0.67)	<0.001	1.064 (0.943,1.2)	0.312	1.535 (1.359,1.735)	<0.001
	Male	0.194 (0.116,0.326)	<0.001	0.615 (0.526,0.721)	<0.001	1.291 (1.119,1.49)	<0.001	1.591 (1.336,1.896)	<0.001
**Hypertension (systolic blood pressure ≥ 140 mmHg or diastolic blood pressure ≥ 90 mmHg or self-report of drug taking)**	Female	0.344 (0.255,0.463)	<0.001	0.372 (0.339,0.408)	<0.001	0.961 (0.889,1.039)	0.314	2.698 (2.49,2.922)	<0.001
	Male	0.344 (0.255,0.463)	<0.001	0.456 (0.416,0.5)	<0.001	1.386 (1.272,1.512)	<0.001	2.312 (2.077,2.572)	<0.001
**Pre-hypertension (120 ≤ systolic blood pressure < 140 mmHg or 80 ≤ diastolic blood pressure < 90 mmHg)**	Female	0.761 (0.575,1.007)	0.056	0.742 (0.656,0.84)	<0.001	1.151 (1.032,1.283)	0.011	1.217 (1.087,1.361)	0.001
	Male	0.761 (0.575,1.007)	0.056	0.96 (0.867,1.062)	0.429	1.114 (1.007,1.233)	0.037	0.949 (0.831,1.083)	0.437
**Ever tobacco smoking**	Female	1.666 (1.355,2.048)	<0.001	1.139 (0.982,1.32)	0.086	0.701 (0.604,0.815)	<0.001	1.23 (1.065,1.421)	0.005
	Male	1.666 (1.355,2.048)	<0.001	1.206 (1.109,1.312)	<0.001	0.783 (0.718,0.853)	<0.001	0.96 (0.86,1.074)	0.483
**Ever cigarette smoking**	Female	1.001 (1.001,1.001)	<0.001	1 (1,1.001)	0.085	0.999 (0.999,1)	<0.001	1 (1,1.001)	0.005
	Male	1.001 (1.001,1.001)	<0.001	1 (1,1)	<0.001	1 (0.999,1)	<0.001	1 (1,1)	0.481
**Current daily cigarette smoking**	Female	1.001 (1.001,1.002)	<0.001	1.001 (1,1.002)	0.057	1 (0.999,1)	0.266	1 (0.999,1.001)	0.663
	Male	1.001 (1.001,1.002)	<0.001	1 (1,1.001)	<0.001	1 (0.999,1)	<0.001	1 (0.999,1)	<0.001

Data in parentheses are 95% confidence intervals (CI).

*Significant at p < 0.05.

**Significant at p < 0.01.

***Significant at p < 0.001.

## Discussion

Based on the first national STEPs survey that ran during the COVID-19 pandemic based on standard protection and safety protocols, we estimated the prevalence of overweight/obesity and distribution of BMI levels in the Iranian population by sex, age, and geographical distribution. Our findings show that, in 2021, approximately 24.96% (24.39–25.53) of the ≥18-year-old Iranian adults were obese and 63.02% (62.39–63.65) were overweight.

We found a significant difference between the prevalence of obesity in male and female participants. The study of the geographical extent of obesity and overweight shows that compared to the national mean, the concentration of weight gain is higher in the northern and northwestern provinces. It is worth mentioning that the lowest estimation belonged to the southeast part of the country. The prevalence of obesity was significantly higher in participants suffering from metabolic and lifestyle risk factors.

However, the predisposing factors and epidemiological patterns follow considerable variation in different populations, and the increasing number of obese and overweight people associated with their adverse health consequences throughout the life course has become an important health priority in many developing countries (2, 4, 12). In 2014, the global age-standardized mean BMI in male and female participants was estimated at 24.2 (24.0–24.4) kg/m^2^ and 24.4 (24.2–24.6) kg/m^2^, respectively. Age-standardized prevalence of obesity was estimated at 10.8% (9.7–12.0) in male participants and 14.9% (13.6–16.1) in female participants. At the same time, 2.3% (2.0–2.7) of male participants and 5.0% (4.4–5.6) of female participants were severely obese (BMI ≥ 35 kg/m^2^) at the global level. The age-standardized global prevalence of underweight was 8.8% (7.4–10.3) in male participants and 9.7% (8.3–11.1) in female participants (3).

Parallel with our findings, several previous studies have presented increasing trends of obesity and overweight in an Iranian population (1, 7, 8, 20, 21). Based on STEPs 2016, with significant difference, the national prevalence of normal weight, obesity, and overweight/obesity in ≥18-year-old Iranian adults was estimated at 36.7% (95% CI: 36.1–37.3), 22.7% (22.2–23.2), and 59.3% (58.7–59.9), respectively (9). Considering differences in sex, the pattern is still higher in women in 2016; obesity prevalence of male participants [15.3% (14.7–15.9)] was higher than female participants [29.8% (29.0–30.5)] (*p* < 0.001) (9).

In 2015, the Global Burden of Diseases (GBD) study revealed that 29.3% of women and 13.6% of male Iranian adults (≥20 years) are obese, significantly lower than our new estimates (20).

There are many medical and nonmedical predisposing factors studied (including age, sex, race/ethnicity, socioeconomic status, and lifestyle patterns) that could be associated with change in BMI levels (2, 22). Given the sex differences, many studies from different countries have shown that, compared to male participants, female participants are at greater risk of obesity (21, 23). Between 1975 and 2016, the age-standardized prevalence of obesity in female participants increased from 10.9% to 33.5% (mean BMI change of 23.3 to 27.3 kg/m^2^) and that in male participants changed from 3.0% to 20.0% (mean BMI change of 21.8 to 25.4 kg/m^2^) (3).

This may be rooted in difference patterns of anatomical fat distribution, fat utilization, or obesity/overweight comorbidities (18). Genetics, sex hormones, and even unknown molecular mechanisms were discussed as probable related factors (17, 18).

From the geographical overview, consistent with previous findings in 2016, the highest levels of BMI belonged to the north and northwestern provinces, which could be associated with their mostly higher economic status (5). Based on a previous investigation, compared to rural areas, the BMI mean was significantly higher in urban areas for all age groups, in both male participants (rural: 24.62, urban: 26.02) and female participants (rural: 26.74, urban: 27.69) (9). Results from other relevant investigations reveal different patterns in increasing the BMI associated with wealth index and income populations (2, 24).

Along with the present findings, previous evidence revealed that participants with metabolic and lifestyle risk factors had mostly increased levels of BMI; previously, the highest levels of BMI were detected in the northwestern and central provinces, which, based on studies, had a mostly higher economic status. Based on the results of the present study, slight changes can be seen in the geographic distribution patterns of overweight and obesity, mainly rooted in changes in lifestyle (9).

Considering the specifications of the COVID-19 pandemic, the design and implementation of the survey with special COVID-19 protection and safety considerations is the greatest achievement of this round of STEPs survey in Iran. This round of survey provides an opportunity to access interaction of NCDs’ risk factors and COVID-19. The present paper, which involves the first national survey during the COVID-19 pandemic, reveals the most updated results of BMI levels and overweight/obesity prevalence by different sex and age groups according to different geographical areas in the country.

Moreover, following STEPwise protocols, the digital online data management and data gathering provided the most reliable data that could lead to accurate evidence for better planning and more effective interventions. We were also faced with many limitations. The cross-sectional nature of this study prevented us from carrying out further inferential analysis. At the same time, data from previous rounds of the STEPs surveys prompted us to do another study using meta-regression to arrive at better trend estimates of BMI or obesity at national and subnational levels (8, 15).

The present study has many important implications. Aligned with many published studies from different countries, our findings confirm that the current ongoing programs and interventions are not enough to stop the rise in BMI (1, 8, 25). The global NCD target of obesity paved the way for global and regional policies for prevention and management of the problem. In order to manage the current situation and to achieve global and national goals, in the field of risk factors and noncommunicable diseases, we must exactly follow our defined national goals (12, 26, 27).

Given the COVID-19 crisis, it should be noted that, compared with normal BMI patients, obese/overweight patients who have COVID-19 are at increased risk for mortality and morbidities. These observations also emphasize the need for increased priority on screening and testing and aggressive therapy for patients with obesity and COVID-19 infections who require more attention and specific planning and interventions (25, 28). As the urgent action, we need to update strategies and action plans that cover intervention for at-risk individuals (25, 28).

It is also worth mentioning that, despite considerable progressive efforts, there are noticeable gaps and limitations in the published evidence required for policymaking. The probable causes of patterns of risk factors, prolonged adverse health effects of COVID-19 in obese patients, and changes in epidemiological transition should be further investigated for various metabolic risks. The findings from related studies suggest that physicians should focus more on COVID-19 patients with obesity as high-risk patients with worse consequences. Monitoring, testing, and an earlier start to vaccination and treatment must also be considered for these groups. On the other hand, changes in behavioral patterns including smoking, physical activity, alcohol intake, and psychosocial factors according to demographic specifications such as sex, age, and ethnicity must be addressed further as complex problems during the COVID-19 pandemic (3, 12, 15, 29).

## Conclusion

To the best of our knowledge, this is the first national STEPs survey conducted during the COVID-19 pandemic based on standard protection and safety protocols. We estimated the prevalence of overweight/obesity and distribution of BMI levels in the Iranian population by sex, age, and geographical distribution. The findings of the present study will help policymakers, clinicians, health executives, and researchers to obtain a more accurate estimation of the obesity/overweight problem and to implement more effective interventional programs.

## Data availability statement

The raw data supporting the conclusions of this article will be made available by the authors, without undue reservation.

## Ethics statement

The studies involving human participants were reviewed and approved by the National Institute for Health Research (ID: IR.TUMS.NIHR.REC.1398.006). The patients/participants provided their written informed consent to participate in this study.

## Author contributions

FF, SD, and SS developed the main design of the manuscript. All co-authors contributed to and participated in the revision of the manuscript. All authors contributed to the article and approved the submitted version.

## Funding

The grant of the study was provided by the Ministry of Health and Medical Education of the Islamic Republic of Iran and the National Institute for Health Research (Grant No. 241/M/9839). 

## Acknowledgments

The authors would like to express their thanks to their partnership with the Deputy for Research and Technology and the Deputy of Health of the Ministry of Health and Medical Education, the National Institute for Health Research, the World Health Organization, and many scholars and experts in related fields. The authors would like to express their appreciation to participants from all over the country who made this experience possible.

## Conflict of interest

The authors declare that the research was conducted in the absence of any commercial or financial relationships that could be construed as a potential conflict of interest.

The reviewer AK declared a shared affiliation with the authors to the handling editor at the time of review.

## Publisher’s note

All claims expressed in this article are solely those of the authors and do not necessarily represent those of their affiliated organizations, or those of the publisher, the editors and the reviewers. Any product that may be evaluated in this article, or claim that may be made by its manufacturer, is not guaranteed or endorsed by the publisher.
